# Translation, Reliability, Psychometric Validation, and Confirmatory Factor Analysis of the Urdu Version of the Self‐Efficacy for Appropriate Medication Use Scale (SEAMS‐U) Among Hypertensive Patients in Lahore, Pakistan: A Validation Study

**DOI:** 10.1002/hsr2.72325

**Published:** 2026-05-03

**Authors:** Muhammad Arshed, Mehwish Kiran, Muhammad Raashed, Ahmad Umar, Somia Bakhtiar Lone, Shafqat Qamer, Muhammad Yaqoob, Sunil Kripalani, Shumaila Zofeen, Shazia Iqbal, Shahzad Ahmad, Muhammad Farooq Umer

**Affiliations:** ^1^ Department of Community Medicine, Baqai Medical College Baqai Medical University Karachi Pakistan; ^2^ University Institute of Public Health, Faculty of Allied Health Sciences University of Lahore Punjab Pakistan; ^3^ Department of Gynaecology and Obstetrics Punjab Employees Social Security Institute Lahore Pakistan; ^4^ Department of Epidemiology and Public Health The University of Veterinary and Animal Sciences (UVAS) Lahore Pakistan; ^5^ Department of Health Professional Technology, Faculty of Allied Health Sciences University of Lahore Punjab Pakistan; ^6^ Department of Basic Medical Sciences, College of Medicine Prince Sattam Bin Abdulaziz University Alkharj Saudi Arabia; ^7^ Center for Health Services Research Vanderbilt University Medical Center Tennessee USA; ^8^ School of Public Health Xi'an Jiaotong University Xi'an Shaanxi China; ^9^ Faculty of Medicine and Health Science The University of Buckingham Buckingham UK; ^10^ Department of Preventive Dental Sciences, College of Dentistry King Faisal University Hofuf Saudi Arabia

**Keywords:** hypertension, medication adherence, SEAMS‐U, self‐efficacy, translation, validation

## Abstract

**Background:**

The Self‐efficacy for Appropriate Medication Use Scale, also known as SEAMS, is a widely used self‐reported measure of confidence in taking medications correctly. However, the scale has not been validated in different languages.

**Aim:**

The present study aims to translate and investigate the psychometric properties of the translated version of the scale SEAMS‐Urdu (SEAMS‐U) administered to hypertensive patients in Lahore, Pakistan, a South Asian country.

**Methods:**

The present study was a cross‐sectional which enrolled hypertensive patients aged ≥ 18 years. The forward–backward method was employed for translation purposes. Content and face validity were assessed, and construct validity was evaluated using principal component analysis (PCA) and confirmatory factor analysis (CFA), with convergent validity also assessed. Internal consistency measures such as Cronbach's alpha, split‐half reliability, and test–retest reliability (over a 4‐week interval) were also employed.

**Results:**

Of the 1012 participants, the majority of our respondents were male (67.4%) and aged 50 and above (45.5%). The SEAMS‐U 13 items had good internal consistency, with Cronbach's alpha: 0.878. In item analysis, Cronbach's alpha varied between 0.849 and 0.883, while the values for the two halves of the questionnaire were 0.838 and 0.789, indicating good reliability of the questionnaire. The test‐retest reliability analysis was adequate (0.686), while the intra‐class correlation (ICC) remained high at 0.814. Exploratory factor analysis (EFA) makes a 2‐factor model in rotated space that explained 68.162% of the total variation. Later, CFA confirmed the 2‐factor model with goodness‐of‐fit measures indicating an acceptable model fit. Convergent validity was adequate, with the Cronbach's alpha reliability of the 9‐item SEAMS‐U 2‐factor model of 0.886.

**Conclusion:**

The SEAMS‐U is a valid and reliable measure of self‐efficacy for antihypertensive medication in Pakistan. This simple tool can be used to assess the confidence of persons with hypertension outside of the tertiary hospital setting to adhere to their medication.

**Abbreviations**
CFA: confirmatory factor analysis
CFI: comparative fit index
CI: confidence interval
EFA: exploratory factor analysis
GFI: goodness of fit index
ICC: intraclass correlation coefficient
IQR: interquartile range
KMO: Kaiser–Meyer–Olkin
MEMS: medication event monitoring systems
NFI: normed fit index
OPDs: outpatient departments
PCA: principal component analysis
RMSEA: root mean square error of approximation
SD: standard deviation
SEAMS: self‐efficacy for appropriate medication use scale
SEAMS‐U: Urdu version of self‐efficacy for appropriate medication use scale
SRMR: standardized root mean‐square residual
TLI: Tucker–Lewis index

## 1 Introduction

Hypertension has emerged as a significant global health challenge in the 21st century, having surged from 594 million to 1.13 billion people globally, with a disproportionate burden falling on low‐ and middle‐income countries (LMICs) [1]. Alarmingly, approximately 1.04 billion individuals with hypertension, constituting 75% of the afflicted population, hail from LMICs [2] with an estimated 8.5 million deaths attributed to elevated systolic blood pressure [3].

Fortunately, evidence suggests that reducing systolic blood pressure can mitigate the risk of cardiovascular events and all‐cause mortality [4]. Even a modest 5‐mmHg reduction in systolic blood pressure corresponds to a 10% decrease in the likelihood of developing cardiovascular events [5]. However, despite the efficacy of antihypertensive medications, achieving optimal blood pressure control remains a formidable challenge, particularly in countries like Pakistan.

Pakistan grapples with hypertension as a pressing public health issue, with an estimated 18% of the adult population affected, rising to 33% among individuals over 45 years [6]. Alarmingly, less than 3% of hypertensive patients in Pakistan have their blood pressure adequately controlled, highlighting a concerning treatment gap [7] and reflecting the pervasive nature of the adherence challenge [8, 9]. Poor medication adherence emerges as a primary culprit behind suboptimal blood pressure management, with a significant proportion of patients failing to adhere to prescribed treatment regimens [10, 11].

The implications of poor medication adherence extend far beyond individual health outcomes, exerting substantial strain on healthcare systems and exacerbating the burden of cardiovascular complications. In the United States alone, medication non‐adherence contributes to 16% of healthcare expenditures, amounting to $500 billion annually [12]. Moreover, individuals with poor adherence face a heightened risk of preventable hospitalizations and re‐hospitalizations, further amplifying the economic toll on healthcare systems [13].

Understanding the complex interplay of factors influencing medication adherence is essential for devising effective interventions. Patients' behaviors regarding medication dosing, timing, and intervals may deviate from prescribed guidelines due to various factors, including health literacy, illness beliefs, and self‐efficacy, especially in rural and underserved areas [14, 15, 16]. Non‐adherence poses a significant barrier to achieving therapeutic goals and optimal health outcomes, whether intentional or inadvertent.

Numerous methods exist for assessing medication use, ranging from traditional pill boxes and calendars to more sophisticated technologies like Medication Event Monitoring Systems (MEMS) and smart blister packs [17, 18]. Despite the availability of diverse measurement tools, there still needs to be a universally accepted gold standard for evaluating adherence [19]. Self‐reported questionnaires, such as the Simplified Medication Adherence Questionnaire and Self‐efficacy for Appropriate Medication Use Scale (SEAMS), offer a cost‐effective and convenient means of assessing medication use across different patient populations and literacy levels [20, 21, 22, 23]. Although the SEAMS has been translated and validated in several languages (e.g., Portuguese, Chinese, Thai, Turkish, and Arabic), no validated Urdu version has been available to date. This gap is particularly significant given that Urdu is one of the most widely spoken languages globally, and the lack of a validated tool limits the ability to assess medication self‐efficacy in a large patient population.

Given its widespread use in healthcare research, this study aims to translate and validate the SEAMS into Urdu, the national language of Pakistan, and to evaluate its psychometric properties among hypertensive patients in resource‐constrained settings.

## 2 Objectives

- To translate the English version of Self‐efficacy for Appropriate Medication Use Scale to the Urdu version (SEAMS‐U).
- To assess the validity and reliability of the SEAMS‐U.
- To assess the Confirmatory factor analysis (CFA) of the SEAMS‐U.

## 3 Methods

### 3.1 Study Design and Setting

This cross‐sectional study was conducted in Lahore, Pakistan, between September 2022 and April 2023. The research was conducted at a public hospital in Lahore, the capital of Punjab, which serves a large and socioeconomically diverse population of hypertensive patients, making it an appropriate setting for validating the Urdu version of the SEAMS. This manuscript was prepared in accordance with the STROBE and COSMIN guidelines, as applicable to observational psychometric validation studies.

### 3.2 Study Participants

The study participants comprised hypertensive patients registered in the hospitals' cardiology and medical outpatient departments (OPDs).

### 3.3 Criteria for Eligibility

Participants were eligible for inclusion if they were adults aged 18 years or older who had been diagnosed with hypertension at least 1 month prior to enrollment and were registered hypertensive patients at the study hospital receiving prescribed antihypertensive medications. Additionally, participants were required to be able to read and communicate in Urdu in order to complete the questionnaire accurately.

Patients were excluded if they had a diagnosed psychiatric disorder, were undergoing planned surgical procedures requiring significant medication adjustments (such as coronary artery bypass grafting or coronary angioplasty), or were experiencing a hypertensive emergency with blood pressure readings exceeding 220/120 mmHg. Pregnant women, lactating mothers, and women within 3 months postpartum were also excluded from the study to avoid potential confounding factors related to physiological changes and medication adjustments. Pregnant women, lactating mothers, and women within 3 months postpartum were also excluded from the study. This exclusion was applied because pregnancy and the postpartum period are associated with physiological changes and pregnancy‐related hypertensive conditions that may require different treatment regimens and medication adjustments, which could potentially confound the assessment of adherence to antihypertensive medications among patients with chronic hypertension.

### 3.4 Participant Recruitment

**Figure 1:**
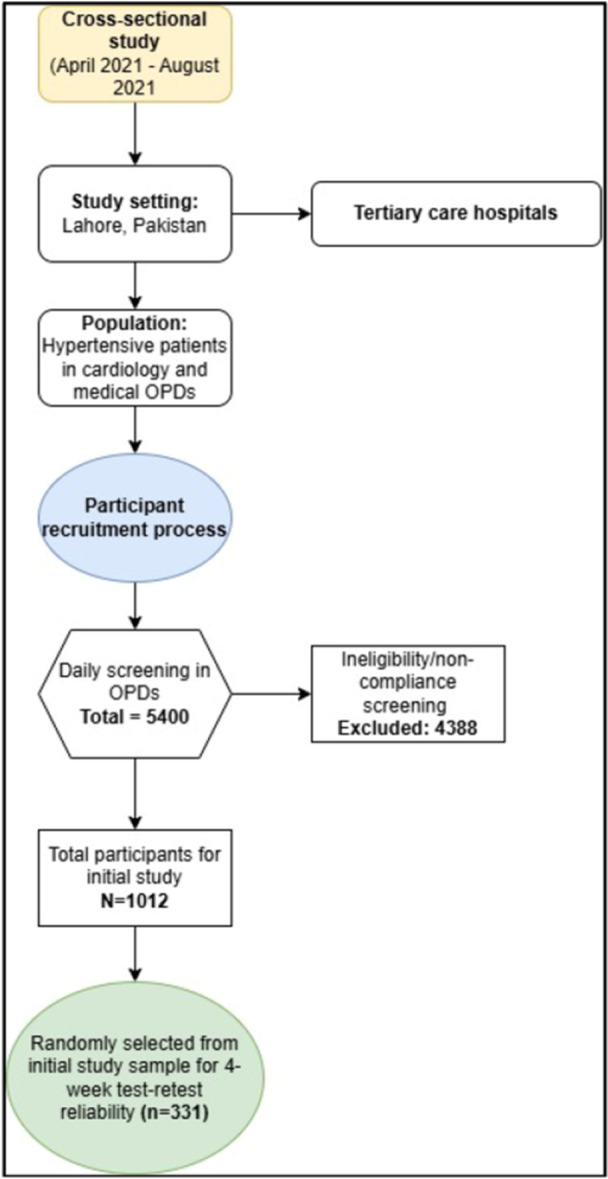
Flow diagram illustrating the sequential steps of participant recruitment, from the total candidate pool to the final recruited participants.

Figure 1 shows the recruitment of participants involved in daily screening in the Cardiology and Medical OPDs of a hospital in Lahore, Pakistan. A designated medical officer, acting as a focal person in each of the OPDs, conducted the screening process for patients who had been hypertensive within the past month. Eligible participants (*n* = 1012) were allowed to select their preferred language, day, and time for interview participation.

### 3.5 Data Collection

The data collection phase encompassed the period from September 2022 to April 2023. Utilizing face‐to‐face interviews, a validated and pretested questionnaire comprising sociodemographic variables and the Urdu version of the Self‐efficacy for Appropriate Medication Use Scale (SEAMS‐U) was administered. Informed consent was obtained from all participants.

### 3.6 Survey Tool

The survey instrument consisted of two sections: (A) sociodemographic and contributing variables; (B) SEAMS‐U‐related scales, administered through face‐to‐face interviews.

At baseline, participants completed a validated questionnaire capturing sociodemographic variables, including age, gender, ethnicity, marital status, education level, economic status, number of daily medications, duration of hypertension, family status, residence, dose frequency, concomitant diseases, use of medication reminders such as alarms, and cigarette use.

The SEAMS, comprising 13 items, assessed participants' confidence levels in medication adherence. Scores ranged from 13 to 39, with higher scores indicating greater self‐efficacy. The SEAMS was translated into Urdu and validated for measuring adherence to hypertension treatment.

### 3.7 Sample Size

Recommendations have been made ranging from 5:1 (a roster of 50 respondents for an instrument with 10 items) to as high as 10:1, even some say up to 15 or, in very rare cases; up to 30. Other recommendations subdivide the sample size into very poor (50 respondents), poor (100), fair (200), good (300), very good, and excellent or more than 1 in a 1000 being best. It is not possible to determine a general rule about the magnitude of sample size required for validation because different types of questionnaires are used and are available in conjunction. In general, larger sample sizes are preferred in psychometric validation studies because they improve the stability and precision of statistical estimates and reduce sampling error. Therefore, efforts were made to recruit a sufficiently large sample to ensure the robustness and reliability of the factor analysis and scale validation procedures. Based on commonly recommended guidelines for factor analysis, which suggest recruiting at least 5–10 participants per questionnaire item, and considering that the SEAMS‐U scale consists of 13 items, the minimum required sample size was estimated to range between 65 and 130 participants. To enhance the stability of the factor structure and improve the reliability of the validation process, a substantially larger sample was targeted and ultimately included in the study [24].

### 3.8 Preliminary Pilot Testing

Like in the questionnaire development process, a pre‐final version of SEAMS‐U was pilot‐tested on 40 intended respondents. The participants were then asked (verbally by an interviewer) to describe what they thought each questionnaire item and corresponding response meant. This iterative process ensures that the translated items maintain their intent of meaning and would help detect misunderstanding in any item among those receiving the questionnaire. The questionnaire translation complement finally resulted after this iteration.

### 3.9 Validity

Questionnaires are examined to see if they measure what is intended to be measured: content validity. It tests not the effects of the questionnaire (whether the inferences, results, and conclusions are built upon them) but whether this space hypothesis can be justified. The Urdu version of the SEAMS was subjected to content, face, and construct validation.

### 3.10 Translating the Questionnaire and Cultural Adaptation

The procedure followed to translate SEAMS into Urdu is summarized in the section below.

The translation and cultural adaptation of the SEAMS scale into Urdu followed a standardized forward–backward translation procedure. Initially, two independent bilingual translators whose native language was Urdu translated the original English version of the instrument into Urdu (forward translation). The two translated versions were then compared and synthesized into a single reconciled Urdu version after discussion among the translators and the research team. Subsequently, this reconciled Urdu version was independently translated back into English by two different bilingual translators who were blinded to the original questionnaire (back translation). The back‐translated versions were compared with the original English version to identify discrepancies and ensure conceptual equivalence. Any inconsistencies were discussed and resolved through consensus to produce the final Urdu version of the scale for use in the study [25]. Following pilot testing and minor refinements based on participant feedback, the final Urdu version of the questionnaire was established for use in the main study.

### 3.11 Expert Committee

It is also recommended that an expert committee prepare a pre‐final version of the translation. The committee was composed of four expert members, which included the original developer expert who was familiar with constructs in questionnaire development; forward and backward translators; and a methodologist. The expert committee then looked at all the translations and judged whether the translated versions had semantic, idiomatic, experiential, or conceptual equivalence with the original. After resolving the discrepancies, consensus was reached by members of the expert committee for each item to prepare a pre‐final version of the translated questionnaire. These results were then used to guide further repetitions of the translation and back‐translation processes.

### 3.12 Data Handling

The data was handled with care prior to conducting any statistical analysis to ensure a smooth process. With only 1% of the data missing, a simple imputation method was employed using the mode for all qualitative variables. This approach preserved the integrity of the dataset while minimizing bias in the analysis.

### 3.13 Statistical Analysis

Normality was assessed using the Kolmogorov–Smirnov test (two‐sided; *p* > 0.05 indicated normal distribution). Item analysis was conducted using item–total correlation (ITC < 0.3), squared multiple correlation (SMC < 0.3), and Cronbach's alpha reliability if item deleted. Test–retest reliability was evaluated using the intra‐class correlation coefficient (ICC) and Spearman's rank correlation coefficient. Construct validity was assessed by Exploratory Factor Analysis (EFA) and subsequently verified by CFA.

Construct validity was assessed by EFA and then verified by CFA [26]. EFA was pre‐specified as the primary analytic approach to evaluate the structural validity of the SEAMS‐U, while CFA was conducted as a confirmatory step. Subgroup and sensitivity analyses were exploratory in nature and are acknowledged as such. To avoid over‐fitting, the total cases were randomly distributed into two equal samples. EFA was employed on first sample, the Kaiser–Meyer–Olkin (KMO) measure (threshold > 0.70) (this test evaluates whether the correlations among the variables are sufficient to justify performing factor analysis). And the Bartlett's test of sphericity (*χ*², two‐sided; *p* < 0.05) was employed to evaluate factorability. Reliability was examined using Cronbach's alpha, split‐half reliability, and ICC. Convergent validity was evaluated through Average Variance Extracted (AVE ≥ 0.5), Composite Reliability (CR > 0.7), and Pearson correlation coefficients (*r* < 0.85; two‐sided; *p* < 0.05).

All statistical tests were conducted at an a priori significance level of *α* = 0.05, and all tests were two‐sided. SPSS (version 27; IBM Corp., Armonk, NY, USA) was employed for descriptive analyses and EFA. CFA was conducted using R Studio (R version 4.3.1; R Foundation for Statistical Computing, Vienna, Austria) with the lavaan package, and the semPlot package was used to generate path diagrams. All statistical analyses were conducted and reported in accordance with the Statistical Analyses and Methods in the Published Literature (SAMPL) guidelines, which provide internationally accepted standards for transparent and accurate reporting of statistical methods in biomedical research.

### 3.14 Ethical Considerations

The research protocol has been approved by the Ethical Committee on Human Research at SAID MITHA teaching hospital, King Edward Medical University, Lahore (5824‐29/E/GSMTH) and the Institutional Review Board of the University of Lahore.

### 3.15 Patient Consent

Informed consent was obtained from all participants after providing a comprehensive explanation of the study's purpose, procedures, risks, and benefits. Participants were assured of the confidentiality of their data and their right to withdraw from the study at any time without any consequences.

## 4 Results

A total of 1012 participants were enrolled in the study, with a response rate of 18.742% from the initially approached participant pool of 5400 who were visiting the OPDs. The relatively high non‐response rate may be attributed to several factors, including limited time availability among patients attending outpatient clinics, reluctance to participate in questionnaire‐based research, ineligibility based on the study criteria, and incomplete questionnaires that could not be included in the final analysis. The age distribution of the selected participants was 45.50% aged 50 and above, 43.32% between 30 and 49, and 11.34% between 18 and 29. Gender distribution indicated a majority of male participants, constituting 67.40% of the total cohort. The ethnic composition showcased diversity, with 67.50% identifying as Punjabi, 21.63% as Suraiki, 8.24% as Urdu speaking, and 2.72% falling into the Others category. Regarding the duration of hypertension, approximately 16.33% reported less than 1 year, 40.90% indicated a duration between 1 and 5 years, and 42.81% reported experiencing hypertension for more than 5 years. Concerning concomitant diseases, 55.14% of participants reported an absence of additional health issues, while 44.92% acknowledged the presence of concomitant diseases. A majority (54.13%) took less than five medications daily, with 33.50% managing between 5 and 9 medications and 12.43% dealing with more than 10 medications daily. In terms of dose frequency, 11.91% adhered to a once‐daily regimen, 80.24% followed a twice‐daily routine, and 7.90% reported a thrice‐daily regimen. Other important sociodemographic characteristics of the selected participants are presented in Table 1.

**Table 1:** Sociodemographic and health‐related characteristics of study participants (*N* = 1012).

Demographics			
		Frequency	%
Age	50 above	460	45.50%
	30–49	438	43.32%
	18–29	114	11.34%
Gender	Female	330	32.63%
	Male	682	67.40%
Ethnicity	Urdu Speaking	83	8.24%
	Punjabi	683	67.50%
	Suraiki	219	21.63%
	Others	27	2.72%
Marital status	Married	806	79.60%
	Single	150	14.84%
	Others	56	5.5%
Socioeconomic status			
Education	Primary and secondary	349	34.50%
	Graduate	291	28.82%
	Post‐graduate	372	36.84%
Family status	Joint family	641	63.31%
	Nuclear family	371	36.70%
Employment	Yes	891	88.02%
	No	121	12.0%
Monthly income	< 10,000	1	0.13%
	10,000–25,000	151	14.90%
	26,000–50,000	240	23.71%
	51,000–100,000	279	27.64%
	> 100,000	341	33.73%
Health‐related characteristics			
Do you smoke?	No	878	86.82%
	Yes	134	13.21%
Duration of hypertension	< 1	165	16.33%
	1–5	413	40.90%
	> 5	433	42.81%
Concomitant disease	No	557	55.14%
	Yes	454	44.92%
No. of daily medications	Less than 5	547	54.13%
	5–9	339	33.50%
	More than 10	125	12.43%
Dose frequency	Once daily	120	11.91%
	Twice daily	811	80.24%
	Thrice daily	80	7.90%

### 4.1 Analysis of Validity and Reliability

#### 4.1.1 Internal Consistency

Cronbach's alpha for the SEAMS‐U 13 items was 0.878 (> 0.7) indicating items are inter‐related to run the EFA. The SEAMS‐U demonstrated average scores ranging from 1.20 to 2.66. Notably, eliminating any scale item would not enhance Cronbach's alpha. The item analysis in Table 2 shows that all other items are meeting the criteria except item 6 and item 12. Both ITC and SMC of these items were less than 0.30 and the Cronbach alpha results increases (> 0.878) if these items were deleted. After removing the item 6 and item 12, the Cronbach's alpha reliability of 11 item SEAMS becomes 0.885.

**Table 2:** Reliability for item analysis of SEAMS‐U.

How confident are you that you can take your medicines correctly?	Mean	SD	ITC	SMC	CA (if Item Deleted)	Item retained
1. When you take several different medicines each day.	2.662	0.561	0.499	0.625	0.873	Y
2. When you take medicines more than once a day.	2.494	0.643	0.601	0.653	0.868	Y
3. When you are away from home.	1.741	0.785	0.479	0.325	0.874	Y
4. When you have a busy day planned.	1.851	0.839	0.779	0.661	0.856	Y
5. When they cause some side effects.	1.314	0.653	0.463	0.591	0.874	Y
6. When no one reminds you to take the medicine.	1.419	0.662	0.297	0.218	0.882	N
7. When the schedule to take the medicine is not convenient.	1.970	0.759	0.726	0.595	0.860	Y
8. When your normal routine gets messed up.	1.674	0.880	0.866	0.821	0.849	Y
9. When you are not sure how to take the medicine.	1.431	0.750	0.560	0.713	0.870	Y
10. When you are not sure what time of the day to take your medicine	1.625	0.807	0.685	0.651	0.862	Y
11. When you are feeling sick (you know, like having a cold or the flu).	1.439	0.729	0.547	0.522	0.870	Y
12. When you get a refill of your old medicines, and some of the pills look different than usual.	1.198	0.492	0.233	0.185	0.883	N
13. When a doctor changes your medicines.	2.565	0.642	0.421	0.307	0.876	Y
Criteria	—	—	> 0.3	> 0.3	≤ 0.878	—

Abbreviations: CA, Cronbach's alpha; ITC, item‐total correlation; SMC, squared multiple correlation.

#### 4.1.2 Test–Retest Reliability

4.1.2.1

Four weeks after the initial assessment, the questionnaire was administered again to evaluate test–retest reliability. A total of 331 participants who had completed the first survey were randomly selected and invited to complete the questionnaire for the second time.

Test–retest reliability was assessed to evaluate the temporal stability of the SEAMS‐U scale. Four weeks after the initial administration of the questionnaire, a subset of participants who had completed the first survey was randomly selected and invited to complete the questionnaire again. A total of 331 participants participated in this follow‐up assessment.

The agreement between the two administrations of the scale was examined using appropriate reliability statistics. The results indicated a high level of consistency between the first and second measurements, demonstrating that the SEAMS‐U scale maintained stable responses over time. These findings support the temporal reliability of the instrument for assessing self‐efficacy in medication adherence among patients with hypertension.

#### 4.1.3 Split‐Half Reliability

The Cronbach's alpha values for the two halves of the questionnaire were 0.838 and 0.789, indicating good internal consistency. The correlation between the two halves was also strong (*r* = 0.783), supporting the reliability of the questionnaire. Both the Spearman–Brown coefficients for equal and unequal lengths of the questionnaire items exceeded the acceptable threshold at 0.878 and 0.879, respectively.

### 4.2 Validity of SEAMS‐U

#### 4.2.1 Content Validity

Content validity refers to the extent to which all of the items in measuring a questionnaire represent the whole construct. It assesses the list of items in the questionnaire following the translation section. When the initial version of the questionnaire was completed, content validity was determined. Content validity of the Urdu version of the SEAMS‐U was evaluated by a panel of experts with relevant expertise in the field. The experts assessed whether the 13 items of the scale adequately represented the underlying construct of medication‐use self‐efficacy and evaluated the relevance and representativeness of each item within its respective domain. Content validation with professionals using the content validity ratio (CVR) and a content validation form was used to quantify expert judgment. The expert panel included a Professor of Public Health and a Professor specializing in English and Urdu linguistics, who reviewed the translated instrument to ensure conceptual accuracy, linguistic clarity, and cultural appropriateness.

#### 4.2.2 Face Validity

Face validity may be measured in terms of lay respondents' recognition that the questionnaire items do measure what they are logically supposed to. This is a lower component of technical judgment and evaluates whether the survey items make sense. While this form of establishing validity is one of the worst, it will at least likely elicit honest answers from participants. The face validity process was conducted in Urdu with laypersons and judges who were hypertensive patients with low‐level education from a public hospital located in Lahore, Pakistan.

#### 4.2.3 Construct Validity

This is a central concept for the assessment of questionnaires, which intends to measure an unobservable construct. A questionnaire without construct validity cannot contribute to drawing inferences, and making interpretations of its results is problematic. Construct validity was analyzed by examining whether the SEAMS‐U scores correlated positively, negatively, or not at all with various items against which they should correlate.

EFA on the first sample (*n* = 506) was performed to assess the scale's structural validity. The KMO value and Bartlett test were looked at to determine the factorability of the sample. After removing the 6th and 12th items from item analysis, the SEAMS‐U 11‐items scale has a KMO value of 0.853 ( > 0.7) and Barlett Test (${\chi^{2}}_{55}$ = 3289.696, *p *< 0.001), indicating that the correlation matrix appropriated to run EFA. In the first run, EFA using PCA and varimax rotation makes a 3‐factor model with by default selection criteria eigenvalue > 1. First factor contained 6 items (3, 4, 7, 8, 9, and 10), second factor contained 3 items (1, 2, and 13), and third factor contained 2 items (5 and 11) with total variance explained by 3 factors, 32.02, 21.11, and 19.63, respectively.

The three‐factor model initially demonstrated acceptable internal reliability, with all factors showing reliability coefficients greater than 0.70 (0.892, 0.793, and 0.766). However, further validation using CFA was considered necessary. The third factor was subsequently excluded for several reasons. First, it contained only two items, whereas factor analysis generally recommends at least three items per factor to ensure stability. Second, item 8, which primarily loaded on Factor 1, also showed cross‐loading with Factor 3 (0.499 > 0.40). Third, the two items within the third factor did not represent a clearly defined conceptual domain.

After removing the 5th and 11th items from item analysis, the SEAMS‐U 9‐items scale has a KMO value of 0.862 (> 0.7) and Barlett Test (${\chi^{2}}_{36}$ = 2580.347, *p*< 0.001), indicating that the correlation matrix appropriated to run EFA. Table 3 shows the results of 2‐factor model if the item 5 and item 11 is removed, the modified SEAMS‐U become reduced to 9‐items with 2 factors. The total variation explained by this model is 68.162% while factor 1 explained 41.55%, and factor 2 explained 26.612% of variations with eigen value 3.74 and 2.40. All factor loading and communalities are greater than 0.40 explaining that good amount of variation is explained by item with corresponding factor and individually respectively. Both factors have at least 3 items.

**Table 3:** Patterns coefficients of exploratory factor analysis with varimax rotation loaded of 2‐ factor model.

How confident are you that you can take your medicines correctly?	Component		Communalities
	F1	F2	*C* ^2^
1. When you take several different medicines each day.	0.157	0.886	0.810
2. When you take medicines more than once a day.	0.309	0.821	0.770
3. When you are away from home.	0.659	0.089	0.442
4. When you have a busy day planned.	0.760	0.360	0.708
7. When the schedule to take the medicine is not convenient.	0.750	0.291	0.647
8. When your normal routine gets messed up.	0.820	0.366	0.805
9. When you are not sure how to take the medicine.	0.816	0.062	0.670
10. When you are not sure what time of the day to take your medicine	0.830	0.175	0.719
13. When a doctor changes your medicines.	0.136	0.738	0.562
Eigen value	3.74	2.40	—
% Variance explained	41.55	26.612	—

Extraction method: principal component analysis.

Rotation method: Varimax with Kaiser normalization.

Rotation converged in three iterations.

**Figure 2:**
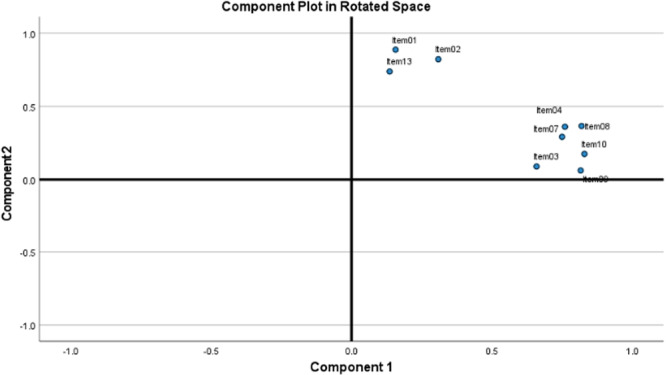
Component plot of 2‐factor model in rotated space illustrating the distribution of items across the first two principal components.

Furthermore, no item showed cross‐loading on other factors. The Figure 2 displays how the rotated two‐factor model formed distinct clusters among the nine items.

The CFA was run on the second sample (*n *= 506) to confirm the 3‐factor or 2‐factor model. Table 4 presents the goodness‐of‐fit indices for the three‐factor model. Overall, the model did not demonstrate an optimal fit. However, some indices, including the Comparative Fit Index (CFI), Normed Fit Index (NFI), and Goodness‐of‐Fit Index (GFI), showed values within the acceptable range. Although the *χ* ^2^/df gave a just acceptable fit, we know that when the sample size is large, the *χ* ^2^ tends to be high. This may lead to rejecting a model falsely and therefore, other fit indices should be employed for model evaluation. For general psychometric techniques, it is recommended to have at least 10 respondents for each item of an instrument, given that SEAMS must have at least 130 respondents for its 13‐item scale. Confirmatory factor analysis was conducted on the nine‐item model using data from 506 participants. Based on the predefined model fit criteria, the *χ*²/df value indicated an acceptable level of model fit rather than an optimal fit.

**Table 4:** Confirmatory factor analysis (CFA) results of goodness‐of‐fit measures for the 3‐factor and 2‐factor model.

Measure	Criteria		CFA Model	
	Acceptable	Good fit	3‐Factor	2‐Factor
χ^2^/df	< 5.0	< 3.0	8.326	3.922
CFI	> 0.90	> 0.95	0.912	0.972
TLI	> 0.90	> 0.95	0.882	0.961
NFI	> 0.90	> 0.95	0.902	0.963
GFI	> 0.90	> 0.95	0.905	0.957
RMSEA	< 0.08	< 0.05	0.120	0.076
SRMR	< 0.08	< 0.05	0.057	0.038

Abbreviations: CFI, comparative fit index; GFI, goodness of fit index; NFI, normed fit index; RMSEA, root mean square error of approximation; SRM, standardized root mean‐square residual; TLI, Tucker–Lewis index.

**Figure 3:**
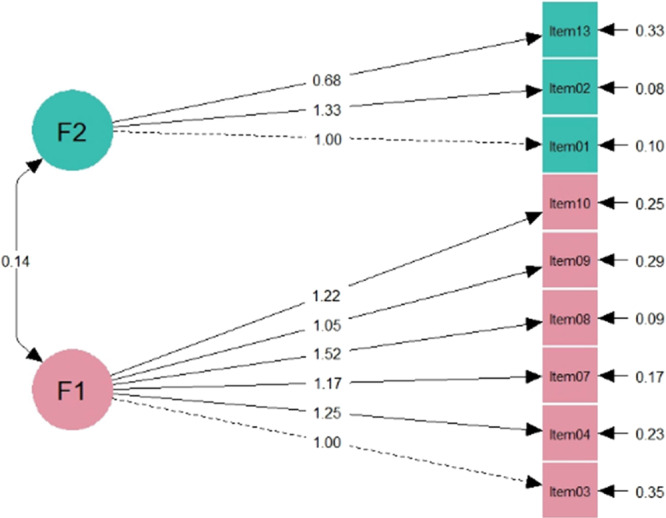
Path diagram of CFA for the 2‐factor model illustrating the relationships between latent variables F1 and F2 and their corresponding observed items. The values on the arrows represent the factor loadings, indicating the strength of the relationship between each item and its respective latent variable. The model highlights the overall fit and complexity of the relationships among the variables.

Figure 3 shows the path diagram of CFA for a 2‐factor model with their estimates of latent and item variables. The model has all significant coefficients and a variance‐covariance structure. Factor loadings indicate the strength of each item's association with the factors, with higher values signifying stronger relationships. The model highlights the overall fit and complexity of the relationships among the variables.

#### 4.2.4 Convergent Validity

Table 5 shows that the factors are validating as the AVE, CR, and CAR in factor 1 (0.60, 0.90, and 0.89), factor 2 (0.667, 0.86, and 0.79), and overall (0.62, 0.94, and 0.886), were above the cutoff (0.5, 0.7, and 0.7), respectively. These results exhibit that the 9‐item SEAMS‐U has an adequate level of convergent validity. The correlation between Factor 1 and Factor 2 was 0.501, which is below the commonly accepted threshold of 0.85. This indicates that the two factors are sufficiently distinct and represent separate constructs. The Cronbach's alpha reliability of the 9‐item SEAMS‐U 2‐factor model was 0.886.

**Table 5:** Convergent validity of the 2‐factor model.

Factors	*N*	AVE	CR	CAR	Correlation
Factor 1	6	0.600	0.900	0.892	0.501*
Factor 2	3	0.668	0.857	0.793	
Overall	9	0.623	0.937	0.886	
Criteria	≥ 3	≥ 0.50	> 0.70	> 0.70	< 0.85

Abbreviations: AVE, average variance explain; CAR, Cronbach's alpha reliability; CR, composite reliability.

* *p*< 0.001.

## 5 Discussion

The SEAMS‐U had good internal consistency. In item analysis, Cronbach's alpha coefficients vary between 0.882 and 0.849, while its split‐half reliability is also good, indicating good internal consistency. The test‐retest reliability analysis was adequate (0.686), while the class correlation coefficient remained high 0.814. EFA make 2‐factor model in rotated space that explained 68.162% of the total variation. Later, CFA confirmed the 2‐factor model with goodness‐of‐fit measures showed the good fit. Convergent validity was adequate with the Cronbach's alpha reliability of the 9‐item SEAMS‐U 2‐factor model was 0.886.

The SEAMS stands out as a valuable and reliable self‐report tool for assessing self‐efficacy in medication adherence, especially suited for patients with chronic health conditions and limited health literacy [20]. SEAMS Urdu version has already been employed to evaluate medication use for hypertension, hyperlipidemia, and other CVDs [22, 27, 28]. Extensively translated and validated in several languages, such as Portuguese, Taiwanese, Thai, Chinese, Turkish, and Arabic, the SEAMS offers a standardized means of evaluating medication use confidence [29, 30, 31, 32, 33, 34]. However, despite its widespread adoption, the Urdu‐speaking population still needs access to a validated scale, despite Urdu being one of the most prevalent languages globally [35]. Consequently, Urdu‐speaking patients have often been deprived of regular assessments of their self‐efficacy for medication use due to the unavailability of suitable measurement tools.

Addressing this gap, our study successfully translated and validated the 13‐item SEAMS into Urdu, demonstrating robust validity and reliability, as evidenced by factor analysis.

In our investigation, the Kaiser–Meyer–Olkin (KMO) measure of sampling adequacy yielded a substantial value of 0.862, indicating a high degree of adequacy for conducting factor analysis on our data set. Our findings regarding the KMO measure align with those reported in previous studies validating the SEAMS in various linguistic and cultural contexts. For instance, the SEAMS Thai version validation study reported a KMO value of 0.67, while the SEAMS Chinese version validation documented a KMO value of 0.828 [29, 32]. Our Bartlett's test result further corroborates the suitability of factor analysis in exploring the underlying structure of medication use confidence among hypertensive patients in Pakistan, underscoring the robustness of our data set.

We determined Cronbach's alpha of 0.886 for the SEAMS scale in our study, signifying a high level of internal consistency reliability. This finding is consistent with previous studies evaluating the SEAMS scale's reliability across diverse linguistic and cultural contexts. For instance, Cronbach's alpha values ranging from 0.88 to 0.931 were reported in the original SEAMS study and other adaptations like the Arabic and Turkish versions, emphasizing the scale's reliability in assessing medication use [30, 31]. The total correlation of corrected material in our study, ranging from 0.28 to 0.82, mirrors findings from prior research, further bolstering the SEAMS scale's robustness and utility in accurately measuring medication use across diverse populations.

Correlation analysis between the test and retest scores of the SEAMS scale revealed a significant positive correlation (Spearman's rho = 0.686, *p* < 0.001), indicating strong consistency in participants' responses between initial and subsequent assessments. These findings resonate with observations from Chinese studies, where the Chinese version of the SEAMS demonstrated reliability, with another study reporting even greater test‐retest reliability [29]. Similar to our findings, the Turkish version of the SEAMS scale exhibited no significant difference between test and retest measures [31], reaffirming the scale's consistency and reliability across diverse populations and languages. Recent studies that employed SEAMS found it to be a reliable instrument for evaluating medication use in chronic illnesses [22, 23, 36].

## 6 Limitations

Despite the strengths of our study, including rigorous validation procedures undertaken for the translated SEAMS‐U, it is essential to acknowledge its limitations. The study was conducted exclusively within a public hospital in Lahore, Pakistan, potentially limiting the generalizability of our findings to hypertensive patients across the entire country. Future research endeavors should include a more diverse sample of patients from various geographic regions and healthcare settings including public and private hospitals, and rural health units to comprehensively understand medication use among hypertensive patients nationwide. Moreover, the eligibility criteria may limit generalizability to some patient subgroups, such as patients with psychiatric disorders, hypertensive emergencies, or pregnant and lactating women. Concerns about the validity of self‐report questionnaires remain unanswered because they are more susceptible to social desirability and recall biases in comparison to other evaluation methods. Our study contributes valuable insights into measuring medication use self‐efficacy among Urdu‐speaking hypertensive patients. It emphasizes addressing linguistic and cultural barriers in healthcare research and practice. We recommend integrating SEAMS‐U into routine outpatient practice in Pakistan to screen for barriers to medication adherence. Training healthcare providers in its use within clinical settings could strengthen hypertension and CVDs management in resource‐limited settings.

## 7 Conclusion

Translating and validating the SEAMS into Urdu is pivotal in assessing medication use among hypertensive patients in Pakistan. SEAMS‐U illustrates good reliability, validity, CFA good fit to the data and CFA for 2‐factor model with their estimates of latent and adequate level of convergent validity. The robust validity and reliability of the SEAMS‐U accentuate its significance in clinical practice and research contexts. With this validated tool, healthcare practitioners gain a nuanced understanding of medication use patterns, enabling them to tailor interventions for improved patient outcomes and mitigated hypertension‐related complications.

The SEAMS‐U is a critical resource in addressing the pervasive issue of poor medication adherence, particularly in low and middle‐income countries. Its implementation facilitates individualized patient care and contributes to broader efforts to enhance the quality of healthcare delivery for hypertensive patients worldwide. As we strive to confront the challenges posed by hypertension on a global scale, the availability of a validated tool like the SEAMS‐U represents a significant stride towards improving health outcomes and reducing the burden of this chronic condition in vulnerable populations.

## Author Contributions

Conceptualization: M.A. Methodology: M.A., M.F.U., M.K., M.R., S.Z., S.A., and S.B.L. Validation: M.A., M.K., M.R., S.Z., S.I., S.A., and S.B.L. Formal analysis: M.A. and S.B.L. Investigation: M.A., M.K., A.U., M.Y., S.Q., and S.Z. Writing – original draft preparation: M.A., M.F.U., M.K., M.Y., S.Q., A.U., and S.B.L. Writing – review and editing: M.F.U., M.K., A.U., M.Y., M.A., S.K., S.Z., and S.B.L. Supervision: M.A., M.F.U., S.Q., M.Y., M.R., and S.Q. All authors have read and approved the final version of the manuscript.

## Funding

The authors have nothing to report.

## Conflicts of Interest

The authors declare no conflicts of interest.

## Transparency Statement

The corresponding authors, Shazia Iqbal and Muhammad Farooq Umer, affirm that this manuscript is an honest, accurate, and transparent account of the study being reported; that no important aspects of the study have been omitted; and that any discrepancies from the study as planned (and, if relevant, registered) have been explained.

## Data Availability

The data will be provided on request by Muhammad Arshed; drarshedchaudhary@gmail.com. The data that support the findings of this study are available from the corresponding author upon reasonable request.
